# Bridging the Gender Gap in Health Care Innovation: The Evolution of FemTech

**DOI:** 10.2196/103487

**Published:** 2026-06-11

**Authors:** Jenny Castillo Cato

**Keywords:** women's health, reproductive health, menstrual cycle, pregnancy, telemedicine

## Abstract

FemTech is a rapidly growing sector in digital health. In this *News and Perspectives* article, JMIR Correspondent and physician Jenny Castillo Cato reports on the evolution and potential of FemTech initiatives for women’s well-being.

**Key Takeaways:**

- Women make up half of the global population yet remain significantly underrepresented in health research and innovation.
- The emergence of FemTech, a sector at the intersection of technology and women’s health care, continues to evolve and expand its offerings, with the potential to transform health care access, advance research, and address systemic gender disparities.

As of 2026, approximately 4.13 billion females make up 49.7% of the global population, with over 21 million adolescent girls in low- and middle-income countries experiencing pregnancy annually. Vaccination rates for human papillomavirus remain low, even though cervical cancer continues to be a major global health issue, with a woman dying every two minutes. As these statistics emphasize, female-focused health care is a major concern worldwide.

Despite this near parity in population and clear need for quality health care, research addressing these issues remains critically underfunded. For example, a 2026 report from the World Economic Forum notes just 6% of private health care investment goes to women’s health, with similar estimates from public funders like the National Institutes of Health in the United States (8.8% of all research funding 2013‐2023) and the Canadian Institute of Health Research in Canada (7.16% of all funded grants in 2023).

While funding is vital for research, female-focused health care is also hindered by cultural obstacles. A review discovered that women’s symptoms are frequently dismissed or erroneously attributed to emotional causes, reflecting deep-rooted gender biases in health care. The combination of the lack of funding and ingrained biases creates an environment where advancement in female-specific health care solutions through traditional means seems nearly impossible. In this landscape, FemTech was born.

## The Emergence of FemTech

FemTech, a term coined in 2016 by Ida Tin, refers to the intersection of technology and female-focused health care. Derived from the previous term “Femcare,” which primarily described menstrual products, Tin repurposed the phrase, broadening it beyond menstruation and the associated taboo, opening it up to a wider audience, and empowering women to take a proactive role in managing their health and well-being.

Initially, FemTech initiatives focused only on menstrual tracking and fertility awareness—areas sometimes referred to disparagingly as “bikini health.” However, over the past decade, innovations in this area have not only improved access to health care resources, but have also helped destigmatize reproductive health and provided women with tools for conception planning and health monitoring. The field continues to expand its applications beyond menstruation, reproductive and sexual health, and pregnancy.

## Drivers of Growth and Change

Several factors have contributed to the rapid evolution of FemTech in North America and increasingly globally, including societal shifts in the perception of women’s health, increased demand, and the increasing digitization of health. Regulatory changes in reproductive rights policy in the United States, including the overturn of Roe v Wade in 2022, also accelerated the demand for telehealth services, particularly for hormone therapy and sexual health care.

Currently, the FemTech world can be broadly categorized into three areas:

1. Clinical services – Telemedicine platforms and personalized health care resources.
2. Engagement solutions – Digital communities and educational applications.
3. Consumer products – Therapeutics, diagnostic tools, and health supplements.

Over the past decade, these offerings have expanded to approximately 4300 companies, with a subset raising US $8.27 billion in funding and spanning a wide variety of focuses. For example, Owlet, founded in 2013, sells wearable baby socks to monitor heart rate and respiration and is considered the top globally funded FemTech company. Another example, Amiya—one of 1337 Ventures’s top 10 FemTech startup winners of 2026—provides emotional support over the in vitro fertilization journey, with both an artificial intelligence companion to help detect emotional distress and connection to a live online community. Curiva, a different type of FemTech innovator, is developing a noninvasive diagnostic patch that uses biomarkers to detect gynecologic cancers following an abnormal Pap smear, potentially reducing the need for invasive procedures such as colposcopies.

The shifting landscape from a narrow focus to much wider catchment of female health care innovations has allowed the FemTech market to expand economically, with global companies increasingly recognizing the importance of the industry. Recently, Qatar Science and Technology Park (QSTP) and Merck created a FemTech Accelerator Program to support up to 30 FemTech startups addressing women’s health issues both regionally and globally. With opportunities like the accelerator program, FemTech is projected to reach a market value of over US $97 billion by 2030 creating a significant global impact.

While FemTech has the potential to improve health care access and outcomes, its technologies can also reinforce existing inequities through biased datasets, culturally insensitive solutions, and approaches that address women’s health issues in isolation rather than within broader systemic contexts. Invocares, a digital health platform for pregnant women, seeks to address these challenges by building a culturally informed platform designed to serve all women, regardless of race, socioeconomic status, or geographic location. Similarly, Health in Her HUE offers culturally competent care, health information, and community support for Black women, women of color, and gender-expansive people of color. By prioritizing inclusivity and cultural responsiveness, these companies exemplify how thoughtful FemTech innovation can help reduce longstanding gender and racial disparities in health care while reshaping how women access and experience medical care.

## The Shift to Deep FemTech

As the funding world starts to catch on, FemTech innovations are moving into their next chapter. “Deep FemTech,” the 2.0 version of FemTech, emphasizes comprehensive health care solutions that extend beyond reproductive health. This shift includes addressing systemic issues such as cardiovascular disease, cancer, and chronic conditions that disproportionately affect women.

**Figure:**
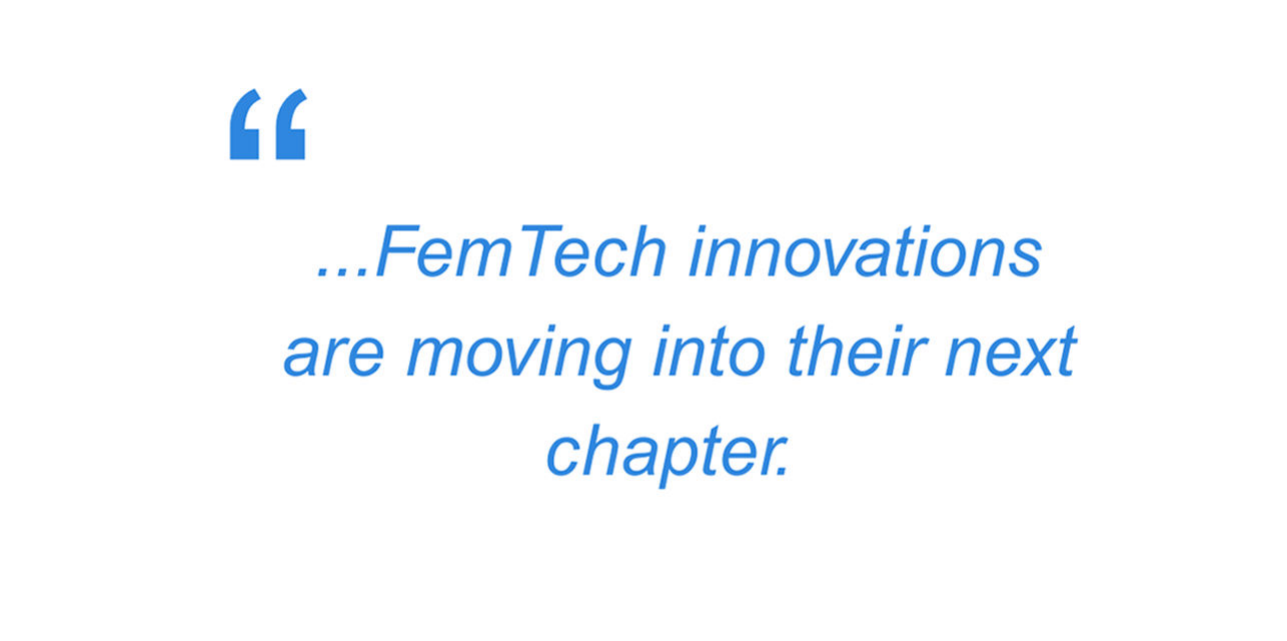


These FemTech innovations represent a transformative movement in health care and an opportunity to address longstanding gender disparities and redefine how women access and experience medical care outside of traditional pathways.

The evolution toward Deep FemTech signals a more inclusive and research-driven future. Continued investment, policy support, and global accessibility will be essential in ensuring that quality health care innovation equitably serves half of the world’s population.

